# Exercise training as a novel primary treatment for localised prostate cancer: a multi-site randomised controlled phase II study

**DOI:** 10.1038/s41598-018-26682-0

**Published:** 2018-05-30

**Authors:** L. Bourke, R. Stevenson, R. Turner, R. Hooper, P. Sasieni, R. Greasley, D. Morrissey, M. Loosemore, A. Fisher, H. Payne, S. J. C. Taylor, D. J. Rosario

**Affiliations:** 1 Health and Wellbeing, Sheffield Hallam University, Sheffield, UK; 2 Acute Therapy Services, Sheffield Teaching Hospitals, Sheffield, UK; 3 Centre for Primary Care and Public Health, Queen Mary University of London, London, UK; 4 Wolfson Institute of Preventive Medicine, Queen Mary University of London, London, UK; 5 William Harvey Research Institute, Queen Mary University of London, London, UK; 6 Institute of Sport Exercise and Health, University College Hospitals, London, UK; 7 Department of Behavioural Science & Health, University College London, London, UK; 8 University College Hospitals, London, UK; 9 Department of Oncology and Metabolism, University of Sheffield, Sheffield, UK

## Abstract

Alternative management strategies for localised prostate cancer are required to reduce morbidity and overtreatment. The aim of this study was to evaluate the feasibility, safety and acceptability of exercise training (ET) with behavioural support as a primary therapy for low/intermediate risk localised prostate cancer. Men with low/intermediate-risk prostate cancer were randomised to 12 months of ET or usual care with physical activity advice (UCwA) in a multi-site open label RCT. Feasibility included acceptability, recruitment, retention, adherence, adverse events and disease progression. Secondary outcomes included quality of life and cardiovascular health indices. Of the 50 men randomised to ET (n = 25) or UCwA (n = 25), 92% (n = 46) completed 12 month assessments. Three men progressed to invasive therapy (two in UCwA). In the ET group, men completed mean: 140 mins per week for 12 months (95% CI 129,152 mins) (94% of target dose) at 75% Hrmax. Men in the ET group demonstrated improved body mass (mean reduction: 2.0 kg; 95% CI −2.9,−1.1), reduced systolic (mean: 13 mmHg; 95%CI 7,19) and diastolic blood pressure (mean:8 mmHg; 95% CI 5,12) and improved quality of life (EQ.5D mean:13 points; 95% CI 7,18). There were no serious adverse events. ET in men with low/intermediate risk prostate cancer is feasible and acceptable with a low progression rate to radical treatment. Early signals on clinically relevant markers were found which warrant further investigation.

## Introduction

Selecting clinical management strategies for men with localised prostate cancer is a balancing act. The evidence in favour of radical treatments such prostatectomy and radiation therapy is disputed, with large randomised studies such as PIVOT and ProtecT demonstrating minimal overall survival advantage^1,2^. Nevertheless, living with an untreated cancer can be a source of anxiety for men^3^ and active monitoring is associated with a higher rate of disease progression and development of metastatic cancer^1,2^. Active surveillance has long been suggested as a primary management strategy for localised prostate cancer, particularly in men thought to be at low risk of progression, however reliably identifying such men is associated with a degree of uncertainly that men and their partners often find difficult to accept^4^, particularly as they perceive no active role in such treatment^5^. Despite the known adverse effects of invasive therapies on quality of life, sexual, urinary and anorectal function^6^, many men opt out of surveillance in favour of what they perceive as an active management strategy involving conventional or investigative ablative therapies.

The benefits of participation in exercise/physical activity and the impact on cancer progression/ mortality outcomes has gained much support from observational data sets describing the impact on solid cancers^7^ and specifically in prostate cancer cohorts^8,9^. Furthermore, a meta-analysis confirmed exercise interventions improve cancer specific outcomes such as disease specific quality of life and physical function as well as reducing fatigue in men with prostate cancer^10^. Pilot ‘lifestyle interventions’ have been attempted in localised prostate cancer. However, it is important to note these studies focused nearly exclusively on modification of ‘lifestyle’ to a vegan diet with soy supplementation, without data on exercise dose/adherence^11^.

In tandem, physical activity will reduce risk of other common cancers and associated co-morbidities such as cardiovascular disease risk, common in elderly populations^12^. Indeed, as demonstrated in the largest RCT thus far comparing treatment options, men with localised prostate cancer are nine times more likely to die of other causes rather than prostate cancer - CVD mortality being nearly three times more likely^1^. Therefore, developing exercise training (ET) as a novel primary treatment option for prostate cancer could fundamentally change the way the disease is managed and offer cancer specific and other health advantages for men affected. The challenge with exercise programmes however, is to support men in starting to become physically active and then maintain exercise over the long-term, whilst providing objective data to confirm behaviour change^13^. Also, such therapies need to be integrated with clinical practice, if they are ever to be considered a realistic treatment option in the health services.

The aim of the Prostate Cancer Novel Therapy (PANTERA) trial was to assess the feasibility and acceptability of delivering ET as a novel primary therapy to men with low and intermediate risk localised prostate cancer with a view to informing a subsequent definitive trial evaluating the effect on progression to invasive therapy.

## Methods

### Study design

The study was a two-arm open label RCT designed and conducted in line with recent updated CONSORT guidance for clinical studies^14^. Following regulatory and ethical approvals (UK NHS, HRA) in accordance with the Helsinki Declaration, 50 men with localised prostate cancer were recruited via outpatient clinics. All men had histological evidence of low or intermediate risk localised prostate cancer, diagnosed using a combination of clinical, bio-chemical, imaging and/or biopsy (confirmed within the previous 12 months) and had elected active surveillance as an initial management strategy^15^. Specifically, only men with Gleason score ≤7 (3 + 4, not 4 + 3), up to T2b clinical stage tumours, with pre-treatment prostate specific antigen (PSA) ≤20 ng/mL and life expectancy of ≥10 years were included. Men with unstable angina, uncontrolled hypertension, recent myocardial infarction (within the past 6 months), pacemakers or those already undertaking regular physical activity (greater than 90 minutes of moderate intensity exercise, per week) or with mental limitation preventing participation in trial assessments, were excluded. All men were recruited from two hospitals in South Yorkshire (UK) and provided informed consent for study participation.

### Randomisation and masking

Randomisation was undertaken using a computer generated algorithm used by cancer prevention trials unit staff at Queen Mary University of London, who were independent of the study team.

### Procedures

#### Exercise training with behavioural support

Aerobic ET was undertaken for 12 months, combining supervised and independent elements. All supervised exercise sessions were guided by a specialist in clinical exercise science and took place at Sheffield Hallam University or community gym facilities involved in the National Centre for Sport and Exercise Medicine network. Men were set a goal of undertaking 150 minutes of exercise per week for 12 months (i.e. 7800 minutes of monitored exercise over 12 months). To support this goal, men were asked to attend two group-based supervised exercise sessions a week, comprising up to 60 minutes of aerobic exercise. Exercise intensity was set at between 65% to 85% of age-predicted maximum heart rate or 12 to 17 on the Borg rating of perceived exertion (RPE) scale^16^, in sessions of 20–30 minutes of continuous exercise for the first 8 weeks, progressing up to 60 minutes per session thereafter. Gym based aerobic ET was conducted using standard ergometers e.g. stationary cycles, rowing ergometers or treadmills. In addition, men were required to undertake self-directed exercise sessions per week, using a heart rate monitor to objectively record independent exercise behaviour to achieve their 150 minute per week target. These men also recorded their exercise behaviour in log books, which were checked on a regular basis.

The behaviour change support was based on the findings of our Cochrane review of interventions to improve exercise behaviour in people living with and beyond cancer^13^. We utilised the following behaviour change techniques as outlined in the CALO-RE behaviour change taxonomy: setting programme goals; prompting generalisation of target behaviour; prompting self-monitoring of behaviour and prompting of practice. Behaviour change counselling conducted by the clinical exercise specialist took place bi-monthly, either via face-to-face sessions during exercise or via telephone for the first three months and then according to participant preference for the following nine months. Ongoing feedback on exercise technique and intensity guidance was provided throughout the supervised sessions as appropriate.

Behavioural support was based on Social Cognitive Theory^17^ (emphasising the importance of self-regulation over willpower) and on Habit Theory^18^ (increases the automaticity of behaviour) arranging, for example, a regular exercise routine with specific times, days and environments to increase the automaticity of physical activity. Decision processes regarding whether to engage in exercise would gradually be replaced by the expectation to engage in exercise, thereby improving maintenance of behaviour change. The habit element aimed to aid the transition to a more active life and, together with increasing self-regulatory skills, to increase the chance of long-term maintenance. Patient perceptions of illness and treatment beliefs were also discussed and addressed with participants using the principals of the necessities and concerns frame work^19^. For example, discussing beneficial and adverse outcomes from participation in active surveillance and undertaking ET as an experimental approach to controlling disease progression. Throughout the 12 months, the clinical exercise specialist helped identify and overcome barriers to exercise, facilitate self-management strategies, review individual exercise goals and promote self-regulatory skills.

### Usual care with exercise advice

Men randomised to usual care with advice (UCwA) underwent active surveillance in accordance with the local cancer network policy, based on the NICE (CG175) recommendations. All men in this group received the Macmillan “Move More” exercise education pack for people living with and beyond cancer (https://be.macmillan.org.uk/be/p-19569-move-more-guide.aspx).

### Outcome measures

The primary outcome was feasibility comprising: recruitment; adherence to ET; study retention: acceptability, adverse event rate^14^ and progression to radical treatment during the intervention period. These data were assessed by extracting data from screening and recruitment logs, attendance at supervised ET sessions as well as independent exercise heart rate recordings, log book records and review of adverse event logs and clinical records. Adverse events were classified according to international ethical, scientific and practical standards i.e. Good Clinical Practice definitions see (https://www.gov.uk/guidance/good-clinical-practice-for-clinical-trials).

Secondary outcomes were assessed at baseline, 3, 6 and 12 months and included resting heart rate (Polar M400, Finland) resting blood pressure and sub-maximal aerobic exercise tolerance assessed by the Bruce treadmill (H/P/Cosmos, Germany) protocol^20^, body mass and BMI. Blood pressure measures were taken by the same clinical investigator throughout with regularly calibrated automated sphygmomanometer (Dash 2500, GE Healthcare, USA) with the average of three recordings taken. Readings were taken in a seated position after the subject had rested for a minimum of 10 minutes. Second and third readings were taken 5 minutes later and the mean pressure of the three readings was recorded as the attendance BP. Quality of life was assessed by the EQ5D questionnaire (see https://euroqol.org/). Dietary habits were assessed by the Food frequency questionnaire^21^. In addition, we assessed the willingness of men to provide one DNA sample for SNP genetic profiling, which could be informative in our planned explanatory trial in terms of predicting response to ET. Other secondary outcomes were safety biomarkers including PSA and serum androgen profile. A lipid profile (taken fasted) and the metabolic biomarker HbA1c was also collected, as requested by patients when we consulted them about study design prior to funding. Samples were processed at local hospital laboratories and reported on the secure internal National Health Service (NHS) results reporting system. Self-reported exercise behaviour was assessed by the Godin Leisure Score index questionnaire^22^ self-efficacy by the SCI Self-Efficacy for Exercise Scale^23^ and exercise habits by the Self-Report Habit Index^24^. Men involved in ET were purposively selected for semi-structured interviews (based on complete vs lower exercise adherence) to offer a qualitative perspective on aspects of acceptability of the intervention and participation in the trial.

### Sample size estimate

Recruitment target was set at 50 men in 12 months. This target assumed that 200 would be screened, 50% of whom would be eligible and 50% of these would agree to participate. For the purposes of future definitive trial planning, the eligibility and participation rates were estimated to within 95% confidence intervals of +/−7% and +/−10% respectively. A 75% study completion rate was estimated to within +/−12%, and a 75% intervention completion rate to within +/−17%. The sample size of 50 was also considered sufficient for estimating the standard deviation of an outcome such as PSA^25^.

### Data analysis

In accordance with the updated CONSORT guidelines for pilot and feasibility studies^14^ outcomes were assessed using standard descriptive methods for rates and proportions. Qualitative methods can be found in online supplement 1.

### Ethics approval and consent to participate

Approved by the UK NHS national research ethics system (reference number 14/LO/0649). All participants provided written informed consent to participate.

### Consent for publication

Participants provided informed consent for the use of anonymised quotes reported in the results section.

### Availability of data and material

The datasets used and/or analysed during the current study are available from the corresponding author on reasonable request.

## Results

### Recruitment /retention

**Figure 1:**
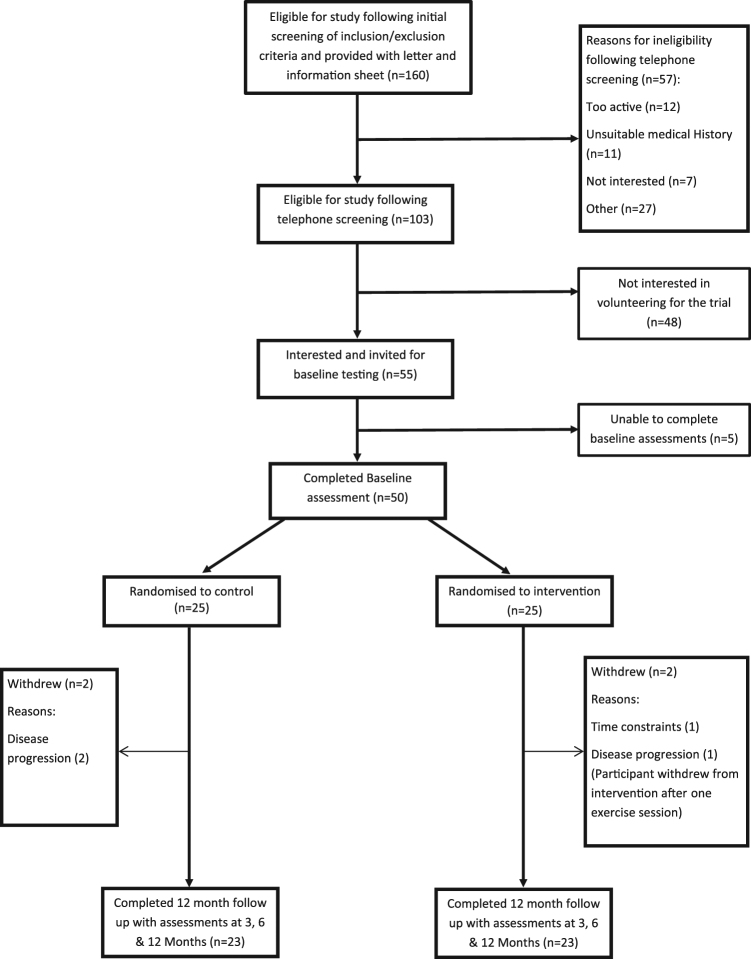
PANTERA trial CONSORT diagram.

From June 2015 to June 2016, we screened 160 men with low to intermediate risk prostate cancer from Sheffield Teaching Hospitals Trust and Barnsley NHS Trust, of whom 103 were eligible for inclusion. Fifty men were recruited and randomised to ET (n = 25) or UCwA (n = 25) (see Table 1 for baseline characteristics). Thus 64% (95% CI 56%,72%) were eligible and 48.5% (95% CI 38.6,58.6) randomised (please see Fig. 1 CONSORT diagram). Retention during the trial was excellent, with 46 men completing 12 month assessments (92%). Progression to invasive treatment over the 12 month follow-up period was equivalent to that seen in a recent large RCT^1^. Overall, three men progressed to radical treatment– two in the UCwA and one in the ET arm.

**Table 1:** Baseline characteristics of men enrolled in the PANTERA study.

	Exercise training (n = 25)	Usual care with advice (n = 25)
Age (yrs)		
mean (SD)	68 (6)	67 (9)
BMI (kg.m^2^)		
mean (SD)	26.7 (2.4)	27.7 (3.2)
Stage (n)		
T1a		1
T1c	14	17
T2a	11	6
T2b		1
Gleason score (n)		
3 + 3	24	23
3 + 4	1	2
PSA (ng.ml^−1^)		
median (range)	5.6 (12.3, 1.2)	5.6 (15.3, 1.4)
Co-morbidities (n)		
CVD	19	12
Joint/bone	26	7
Metabolic	5	2
GI	7	5
Chronic pain	1	3
Mental health		3
Pulmonary	1	3
Eye disorder	2	
Inner ear disorder	3	
Skin disorder	1

### Exercise training adherence

**Figure 2:**
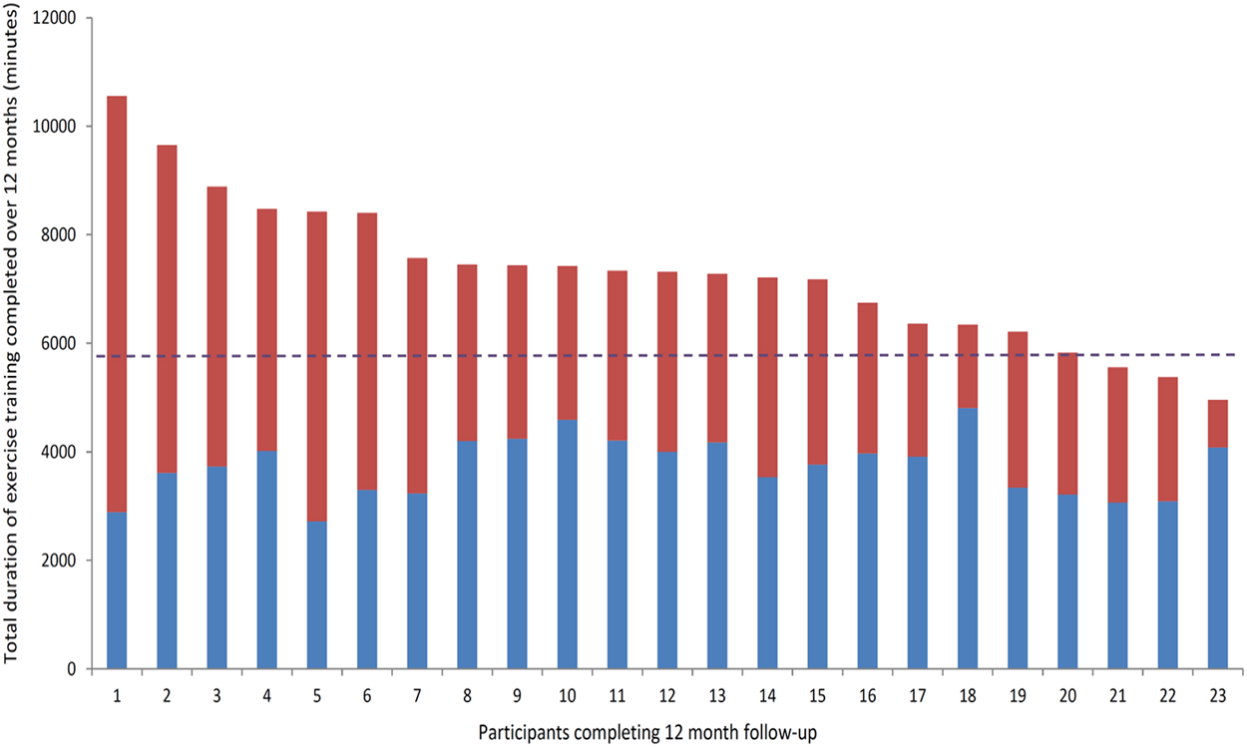
Total exercise behaviour over 12 months in minutes for all 23 exercise training participants, with blue bars = supervised exercise and red bars = independent exercise. Dashed line = 75% adherence or 5850 minutes in total.

Adherence in the ET group was excellent with an average of 7304 min (95% CI 6719,7890) completed at 75% Hr max (i.e. 94% of target), or 140 min on average per week (95% CI 129,152) over 12 months. This was broken down as 3725 min supervised exercise (95% CI 3487,3964) at 77% Hr max (or RPE of 12) and 3579 min of independent exercise (95% CI 2911,4247) at 73% Hr max in total over 12 months (please see Fig. 2).

### Adverse events

No serious adverse events were reported over the duration of the trial. A total of 64 adverse events were recorded: only 8 were related to participation in the ET or study assessments and involved minor chest discomfort, mild joint and soft tissue pains and one instance of a nose bleed (pre-existing condition).

### Secondary outcomes

Large, clinically relevant^26^ effect sizes were seen in the ET group in terms of reduction in diastolic (mean: 8 mmHg; 95% CI 5,12) and systolic blood pressure (mean: 13 mmHg; 95% CI 7,19) along with substantial improvements in submaximal fitness (mean:171 s; 95% CI 124,219), body mass (mean: −2.0 kg; 95% CI −2.9, −1.1), Godin LTI questionnaire score (mean:20 points; 95% CI 12,27) and EQ5D questionnaire score (mean:13 points; 95% CI 7,18). An increase in UCwA group self-reported exercise behaviour on the Godin questionnaire over the 12 months was also observed (mean:15 points; 95% CI 5,25) however this had only a small impact on physiological and fitness outcomes such as submaximal fitness, diastolic and systolic blood pressure: please see Table 2. Data from safety biomarkers (PSA, serum testosterone, sex hormone binding globulin, free androgen index) indicates minimal changes in biochemical metrics in both groups: please see Table 3.

**Table 2:** Secondary outcomes from the trial.

	Resting Hr (b.m^−1^)	Diastolic BP (mmHg)	Systolic BP (mmHg)	Sub-maximal fitness (s)	Body mass (kg)	BMI (kg/m^2^)	Physical activity^£^	Quality of life^$^
**Usual care with advice**								
**Baseline**								
Mean	66	77	139	525	81.1	26.7	14	71
95% CI	(59, 73)	(73, 82)	(132, 146)	(461, 588)	(77.4, 84.9)	(25.6, 27.7)	(10, 18)	(64, 78)
**3 months**								
Mean	62	76	136	580	80.9	26.7	18	76
95% CI	(56, 69)	(73, 80)	(130, 143)	(537, 623)	(77.1, 84.7)	(25.6, 27.9)	(15, 22)	(70, 81)
**6 months**								
Mean	59	76	134	581	80.4	26.6	21	77
95% CI	(54, 64)	(72, 79)	(128, 140)	(528, 634)	(76.9, 83.9)	(25.6, 27.6)	(16, 27)	(72, 83)
**12 months**								
Mean	61	76	135	574	80.4	26.6	29	79
95% CI	(56, 65)	(72, 79)	(127, 142)	(523, 625)	(76.8, 84.0)	(25.5, 27.7)	(19, 40)	(73, 85)
Change*	−5	−1	−4	49	−0.7	−0.1	15	8
	(−11, −1)	(−5, 2)	(−10, 2)	(18, 81)	(−2.2, 0.7)	(−0.5, 0.5)	(5, 25)	(1, 16)
**Exercise training**								
**Baseline**								
Mean	68	82	145	470	84.5	27.7	23	71
95% CI	(62, 74)	(79, 85)	(138, 152)	(395, 544)	(80.7, 88.3)	(26.4, 29.1)	(16, 31)	(64, 79)
**3 months**								
Mean	64	77	136	569	84.4	27.7	35	76
95% CI	(59, 69)	(74, 80)	(129, 142)	(508, 630)	(80.3, 88.4)	(26.2, 29.1)	(19, 50)	(70, 82)
**6 months**								
Mean	64	76	133	603	83.4	27.4	37	79
95% CI	(59, 68)	(73, 79)	(126, 139)	(545, 662)	(79.9, 87.4)	(26.2, 28.7)	(29, 45)	(74, 83)
**12 months**								
Mean	62	74	132	641	82.5	27.1	43	84
95% CI	(58, 66)	(71, 77)	(125, 139)	(579, 703)	(78.7, 86.3)	(25.7, 28.5)	(33, 53)	(80, 87)
Change*	−6	−8	−13	171	−2.0	−0.6	20	13
	(−10, −3)	(−12, −5)	(−19, −7)	(124, 219)	(−2.9, −1.1)	(−0.9, −0.4)	(12, 27)	(7, 18)

Data is from 46 men completing 12 months follow-up. *Mean difference between baseline and 12 months with 95% confidence intervals. ^£^Exercise behaviour measured by the Godin questionnaire, ^$^Quality of life measured by the EQ5D questionnaire. Submaximal fitness was measured by Bruce treadmill protocol, data is from 42 complete cases over the four trial assessments.

**Table 3:** Safety and other biomarkers from baseline to 12 months of follow-up.

	PSA(ng/ml)	ST (nmol/L)	SHBG (nmol/L)	FAI	LDL (mmol/L)	HDL (mmol/L)	Total C (mmol/L)	TRIGs (mmol/L)	HbA1c (mmol/mol)
**Usual care with advice**									
**Baseline**									
Mean	5.8	18.0	51.4	34.1	3.1	1.5	4.8	1.1	37.8
95% CI	(4.7, 6.9)	(15.2, 20.8)	(45.5, 57.4)	(30.0, 38.1)	(2.5, 3.6)	(1.3, 1.6)	(4.2, 5.4)	(0.9, 1.3)	(35.4, 40.3)
**3 months**									
Mean	5.6	16.9	55.2	31.7	2.9	1.5	4.9	1.0	37.0
95% CI	(4.6, 6.6)	(14.3, 19.5)	(46.9, 63.5)	(27.8, 35.6)	(2.4, 3.4)	(1.4, 1.7)	(4.3, 5.4)	(0.8, 1.2)	(34.9, 39.1)
**6 months**									
Mean	5.9	18.5	57.2	34.1	2.8	1.5	4.9	1.2	37.2
95% CI	(4.7, 7.1)	(16.1, 21.0)	(48.8, 65.6)	(29.6, 38.7)	(2.4, 3.3)	(1.3, 1.7)	(4.3, 5.4)	(0.9, 1.5)	(34.8, 39.7)
**12 months**									
Mean	5.7	18.1	56.5	31.4	2.9	1.5	4.9	1.1	36.4
95% CI	(4.5, 6.9)	(15.7, 20.5)	(49.7, 63.3)	(27.6, 35.1)	(2.4, 3.3)	(1.4, 1.7)	(4.4, 5.4)	(0.9, 1.2)	(34.9, 38.0)
Change*	−0.1	0.1	5.1	−2.7	−0.2	0.0	0.1	0.0	−1.4
	(−1.0, 0.7)	(−1.7, 1.8)	(1.8, 8.3)	(−5.8, 0.4)	(−0.4, 0.0)	(0.0, 0.1)	(−0.2, 0.3)	(−0.2, 0.2)	(−3.0, 0.2)
**Exercise training**									
**Baseline**									
Mean	6.4	14.3	44.2	33.2	2.6	1.4	4.5	1.3	40.9
95% CI	(5.1, 7.7)	(12.3, 16.4)	(37.6, 50.8)	(29.1, 37.3)	(2.2, 3.0)	(1.2, 1.5)	(4.0, 5.0)	(1.0, 1.6)	(36.6, 45.2)
**3 months**									
Mean	6.3	14.5	46.2	31.3	2.5	1.4	4.4	1.3	40.4
95% CI	(5.1, 7.5)	(12.6, 16.4)	(38.2, 54.3)	(26.8, 35.8)	(2.0, 2.9)	(1.2, 1.5)	(3.9, 4.9)	(1.0, 1.7)	(36.2, 44.6)
**6 months**									
Mean	6.1	13.9	46.0	32.0	2.5	1.3	4.4	1.3	38.8
95% CI	(4.9, 7.3)	(12.1, 15.7)	(37.8, 54.2)	(27.7, 36.4)	(2.1, 2.9)	(1.2, 1.5)	(4.0, 4.8)	(1.0, 1.5)	(34.8, 42.8)
**12 months**									
Mean	6.4	14.2	48.7	31.0	2.3	1.4	4.3	1.3	41.8
95% CI	(5.0, 7.9)	(12.2, 16.2)	(40.2, 57.3)	(27.1, 35.0)	(1.9, 2.7)	(1.2, 1.5)	(3.9, 4.7)	(1.0, 1.7)	(36.0, 47.6)
Change*	0.0	−0.1	4.5	−2.2	−0.3	0.0	−0.2	0.0	0.9
	(−0.6, 0.7)	(−1.6, 1.3)	(1.2, 7.8)	(−5.3, 1.0)	(−0.5, −0.1)	(−0.1, 0.1)	(−0.5, 0.0)	(−0.2, 0.3)	(−2.2, 4.0)

PSA = prostate specific antigen, ST = serum testosterone, SHBG = sex hormone binding globulin, FAI = free androgen index, LDL = low density lipoprotein, HDL = high density lipoprotein, Total C = total cholesterol, TRIGs = triglycerides, HbA1c = glycated haemoglobin. *Mean change between baseline and 12 months of follow-up. Data is from 46 men completing 12 months follow-up.

Over the 12 months follow-up men randomised to ET reported increases in self-efficacy (mean change: 2 points; 95% CI 0, 4) and automaticity of behaviour (mean change: 6 points; 95% CI 2, 10). Men randomised to UCwA also reported increases in automaticity of behaviour (7 points; 95% CI 2, 12) and self-efficacy (mean change: 1 point; 95% CI −2, 4). We asked men to complete food frequency questionnaires at baseline and 12 months, however, the feasibility of collecting such data using this tool proved problematic, with only 21 patients returning both baseline and 12 month questionnaires. The feasibility of collecting DNA samples for SNP analysis was good with 49/50 men agreeing to provide a sample. Analysis on DNA SNPs will be presented elsewhere.

### Acceptability and qualitative data

Qualitative feedback from men who took part in the ET is presented in four superordinate themes and twelve subthemes were identified (Table 4). These themes are complemented by selected quotes below (see online supplement 1 for full details).

**Table 4:** PANTERA qualitative feedback organised into superordinate and subordinate themes will illustrative quotes.

Superordinate themes	Subordinate themes	Selected illustrative quotes
1. Motivations for participation in the trial	1.1 Management of prostate cancer	“*Well he told me I’d got this stage one prostate cancer, and that by doing exercises it could help reduce it, reduce the PSA, so that’s why I was involved with it”. 159*
	1.2 To benefit others in the future	“*Well, I do believe that, you know, that people should, should do trials and was quite willing to do trials if it’s going to help myself and mainly if it’s going to help others in the future”. 469*
	1.3 Improvement of fitness	“*I wanted to get fit, because I was very unfit”. 205*
	1.4 Initial concerns	“*It was something, I hadn’t gone to a gym before, so I got a bit worried at the first couple of sessions, but I was all right then”. 159*
2. Trial design	2.1 Delivery	“*Actually it all right, it went very quickly. And had it said do another six months or another year I’d have done it. Yeah, because I was enjoying it and it was somewhere, I mean once you’re retired you look forward to going to certain places, and that’s what I did. I enjoyed it“. 159*
	2.2 Intensity	“*No, it was about right. I mean I did start doing a bit more as I went on, and obviously you improve over that period of time anyway so you go a bit faster or work a bit harder. So yeah, I found it quite within my capabilities”. 310*
	2.3 Monitoring	“*Yeah, my generation are being left behind with technology basically. And even if it’s just a simple question of pressing the right button, you don’t always find it straightaway. But essentially I mean I would say I was 90–95% of the time it’s fine”. 248*
3. Adherence	3.1 Supervision with integrated behavioural support	“*If I’d been left unsupervised I probably would have kept on the same levels of machinery; whereas he urged us to go forward a bit higher each time. And we got on quite well, he was very good”. 159*
	3.2 Perceived benefits of peer support	“*The, thought that other people on the group were probably going to be there and that I was missing out”. 442*
	3.3 Psychological and physical benefits	“*Yeah I did, I think it does help with mental issues anyway. People with depression and things like that I think it helps. And I think a lot of people were cheesed off with it, with prostate cancer”. 205*
	3.4 Flexibility	“*Yeah, yeah and like I said if anything else happened I’d, there were always alternative times, so if ought happened in the morning and I weren’t able to go, there was always another, he’d always got another alternative for you”. 202*
4. Impact	4.1 Quality of life	*There’s a few, I mean obviously physically I think I’m fitter than I’ve ever “been! Um, so I think it’s been, you know, health, health wise in that just feeling better and feeling fitter has been good and I think there is a bit of a, it’s quite supportive to go to something with other people who are, who have got the same issues”. 447*
	4.2 Health improvements	“*I know I’d, you know, I’d, I’d wrote some things down as we started the course about losing weight, getting fitter and that and so I were able to, and that’s what happened, you know, lost my weight”. 202*
	4.3 Confidence	“*To be honest what the gym did, it gave me, I mean I was unfit but it gave me the confidence to do it”. 205*
	4.4 Improvement in exercise levels and behaviour	“*I have joined a gym; I go three times a week now”. 159*

### Motivations

Being diagnosed with prostate cancer and being approached to take part in this trial was an incentive for taking part. It was initially discussed with the men that this trial may be of some benefit to their disease and other aspects of their health, the men felt that participation would help them achieve these suggested benefits.

“*Well anything that would help you in a desperate situation like having cancer, I mean I appreciate that I’m a low risk at the moment. That’s the most, that’s all the motivation I needed really. And then as you get into it you realise that it’s therapeutic in all sorts of ways”*. (248)

### Adherence

The trial demonstrated excellent adherence rates (94%) and the reasons for the men’s adherence was discussed in great depth. They reported that supervision and having behaviour change support integrated into the supervision was a crucial factor in adherence.

“*I think a combination. It does make you more likely to attend, and also gives you a bit more confidence that somebody’s looking over you if you like”*. (248)

Some of the men had previous experience of other exercise referral schemes such as cardiac rehabilitation, which lacked behaviour change support, with the men stating why this trial was different and more engaging than previous experiences.

“*Well, yeah, I have to say the whole programme was based around goal setting, because not only did we do the exercise in the gym, but we also sat with the specialist from time to time and reviewed our, or set our goals and reviewed our progress towards meeting those goals and we talked about rewards and all that kind of thing, which never happened on the initial cardio programme”*. (202)

### The trial

Practicalities of the trial were discussed. The majority of the men though the duration and frequency of the trial was sufficient, with some stating they would have continued having supervised sessions longer than 12 months due to benefit and enjoyment. The delivery sites were seen as being convenient to men; however, parking was sometimes an issue.

“*Actually it all right, it went very quickly. And had it said do another six months or another year I’d have done it. Yeah, because I was enjoying it and it was somewhere, I mean once you’re retired you look forward to going to certain places, and that’s what I did. I enjoyed it”*. (159)

### Impact

The men discussed how the programme had impacted upon their quality of life, fitness levels, physical and mental health. Men reported better improved fitness, ability to engage in everyday physical tasks without struggling as much as before and an improved mental health.

“*I have to say the, the programme; the PANTERA programme’s transformed my life”*. (442)

“*I know I’d, you know, I’d, I’d wrote some things down as we started the course about losing weight, getting fitter and that and so I were able to, and that’s what happened, you know, lost my weight”*. (202)

## Discussion

The PANTERA trial is the first multi-centre RCT to report the feasibility of aerobic ET as primary therapy for men with localised prostate cancer with retention of men randomised to ET extending to 12 months. Our study demonstrated good feasibility and acceptability in terms of recruitment, retention, sustained behaviour change and adherence with few adverse events and important impacts on clinically relevant outcomes in this population of men with localised disease.

Observational data has suggested that physical activity could improve prostate cancer–specific mortality and overall mortality^8,9^. Few clinical studies to date have evaluated the potential of ET as direct anti-cancer treatments in men with prostate cancer. A small single centre RCT of 26 men with localised disease recently reported feasibility outcomes from a combined lifestyle intervention of exercise and whole-grain rye diet supplementation^27^. Over a six month intervention, the authors reported a median level of 91 min/week of vigorous activity for the first 3 months and 66 min/week for the last 3 months from heart rate monitor recordings. It is important to note this study reported an adverse ratio of prostate cancer progression of 3 vs 0 men from the intervention group vs controls during the trial. Further this study retained less than 75% of its cohort, excluded men with PSA above 10 and also those with moderate or severe co-morbidity. The present study performed better in terms of trial retention, objectively recorded exercise behaviour, had much more inclusive enrolment criteria (see Table 1) and over a 12 month period, three men progressed to radical treatment: only one doing so from the ET group after discontinuing with exercise very early in the programme (i.e. after just one session).

The mechanisms whereby aerobic exercise reduces PCa progression are not clear: several pathways around how exercise might interact with the tumour microenvironment have been covered in a recent review^28^. Nevertheless, the ‘dose’ of exercise required to achieve benefit has only been estimated from observational data. Pilot ‘lifestyle interventions’ have been attempted but focused almost exclusively on strict dietary modification i.e. to veganism, without data on exercise dose/adherence^11^. In contrast, we have used a specific and pre-specified target exercise intensity, supported by regular supervision and goal-setting:^13^ the majority of men were able to either reach or indeed exceed such a threshold. Future large-scale trials are required to establish the dose-response curve and relationship between aerobic exercise and progression of localised prostate cancer. Encouraging data from a small single centre RCT in men with biochemical recurrence following radical prostatectomy has reported improvements in fitness levels correlated with increasing PSA doubling time (n = 12 completing training)^29^. We saw no impact on PSA over our 12 month follow-up, but a longer follow-up could elucidate biomarker responses associated with cancer progression using ET as prescribed and delivered in PANTERA.

The matter of acceptability of ET within active surveillance should be mentioned. Although self-empowerment has received much attention in cancer management in recent years, most of the effort and literature revolves around survivorship or self-empowerment *after* definitive management. Men with prostate cancer often describe low levels of empowerment. Given the controversy around the risks and benefits of invasive therapies for localised prostate cancer, and the potential benefit suggested in reducing disease progression and mortality from observational studies of physical activity, there is a real imperative to provide prospective evidence for such a management strategy. Qualitative evaluation within this study found support from participants for continuing exercise prescription and longer follow-up (see online supplement 1).

One of the commonest causes of death in men with prostate cancer is cardiovascular disease. Indeed, as demonstrated in the largest RCT thus far comparing treatment options, men with localised prostate cancer are nine times more likely to die of other causes rather than prostate cancer - CVD mortality being nearly three times more likely^1^. As such men have a diagnosis of cancer, in the absence of autopsy data or central review of cause of death, it is possible that the quoted rates are an underestimate in clinical practice. Physical activity is associated with reduced risk of cardiovascular mortality in the general population as well as a reduction in the incident risk of other common cancers^12^. We also found the ET impacted important markers of cardiovascular health i.e. both systolic and diastolic blood pressure in our study cohort. Whilst these are not cancer-specific, these represent clinically relevant changes^26^ in important health outcomes in elderly men. The cardio-protective value of ET as a pre-cursor to conventional cardio-toxic prostate cancer treatments such as ADT^30^ is something of real clinical and health-economic interest in the management to prostate cancer survivors.

Estimated costs of ET per-man in the PANTERA study would be £855 p.a. inclusive of specialist supervision, behaviour change, access to a community exercise facility and four progress/progression assessments per year. This cost figure does not take into consideration any health economic benefits from the ET such as fewer invasive cancer treatments, the cost to the health service of treating adverse effects of those treatments or reduction in risk of new co-morbidities associated with age. ET also provided qualitative evidence about improved feelings of wellbeing which is supported by data from EQ5D assessments reporting improved quality of life. This is consistent with trials designed to improve cancer-specific quality of life in men with advanced disease^10^. This is in stark contrast to conventional invasive approaches to localised disease management which have well established adverse effects on sexual, urinary and anorectal function^6^. Therefore, developing ET as a novel primary treatment option for prostate cancer could fundamentally change the way the disease is managed and offer cancer specific and other health advantages.

There are limitations to the present study that should be noted. Follow-up will likely need to be much longer in our definitive trial, extended to at least 3–5 years to provide sufficient time to detect changes in progression to invasive treatment endpoints. No blinding was used in this phase II trial, so preliminary effects on subjective outcomes need to be interpreted with caution. More acceptable methods of collecting dietary confounders will need to be included in subsequent studies (e.g. three day diet diaries), given the food frequency questionnaire was poorly completed in our present cohort. Mechanistic data would be helpful in interpreting progression free survival data in the subsequent study: this will likely have to extend beyond typical kallikrein markers and could include immunological, metabolic and inflammation markers as well as circulating tumour cell profiles. Whilst the study was open to men with PSA over 10 ng.ml^−1^ and Gleason score above 6, the majority of recruits had a PSA of less than 10 ng.ml^−1^ and Gleason score 3 + 3. Strategies to recruit men with intermediate risk disease could be developed to inform our definitive trial. The inclusion of exercise advice in the usual care arm could be a potential confounder in determining the true impact of a supervised exercise intervention. Nevertheless, given the observational evidence supporting the benefits of exercise in such an age cohort as well as the predominantly sedentary nature of the men recruited, providing no exercise guidelines was seen as unethical in this group. In any case, the provision of such guidance to the usual care group could have the effect of reducing the differences in study outcomes between the groups, something that will need to be factored into any future definitive study.

## Conclusion

The PANTERA study demonstrated encouraging feasibility and early signals on clinically relevant markers in men with prostate cancer as a result of ET. A multi-centre definitive clinical trial to establish clinical and cost effectiveness is now indicated before such interventions could be considered as part of management in clinical practice.

## Electronic supplementary material

Supplementary Dataset 1
