# Supplementary material for: Exercise training as a novel primary treatment for localised prostate cancer: a multi-site randomised controlled phase II study
Source: Sci Rep. 2018 May 30;8:8374. doi: 10.1038/s41598-018-26682-0 (PMC5976628; doi:10.1038/s41598-018-26682-0)
Supplement: Supplementary file 1 — Supplementary Dataset 1 [file 41598_2018_26682_MOESM1_ESM.docx]

**Title**: Exercise training as a novel primary treatment for localised prostate cancer: a multi-site randomised controlled phase II study.

**Authors**: Bourke L,^1^ Stevenson R,^2^ Turner R,^1^ Hooper R,^3^ Sasieni P,^4^ Greasley R^1^, Morrissey D,^5^ Loosemore M,^6^ Fisher A,^7^ Payne H,^8^ Taylor SJC,^3^ Rosario DJ.^9^

**Affiliations**:

1. Health and Wellbeing, Sheffield Hallam University, Sheffield UK.

2. Acute Therapy Services, Sheffield Teaching Hospitals, Sheffield, UK

3. Centre for Primary Care and Public Health, Queen Mary University of London, London UK

4. Wolfson Institute of Preventive Medicine, Queen Mary University of London, London UK

5. William Harvey Research Institute, Queen Mary University of London, London UK

6. Institute of Sport Exercise and Health, University College Hospitals, London, UK

7. Department of Behavioural Science & Health, University College London UK

8. University College Hospitals, London, UK

9. Department of Oncology and Metabolism, University of Sheffield, Sheffield UK.

**Key words**: Prostate cancer, aerobic exercise training, feasibility, localised disease, active surveillance.

**Trial identifier**: NCT02409212, registered April 6^th^ 2015.

**Corresponding author**: Professor L Bourke. Office Q103, Parkholme, Sheffield Hallam University, S10 2BP. email [l.bourke@shu.ac.uk](mailto:l.bourke@shu.ac.uk) Telephone +441142255396

**Running head**: Exercise training as primary therapy for localised prostate cancer

**Emails**: [l.bourke@shu.ac.uk](mailto:l.bourke@shu.ac.uk); [Richard.Stevenson@sth.nhs.uk](mailto:Richard.Stevenson@sth.nhs.uk); [Rebecca.Turner@shu.ac.uk](mailto:Rebecca.Turner@shu.ac.uk); [r.l.hooper@qmul.ac.uk](mailto:r.l.hooper@qmul.ac.uk); [p.sasieni@qmul.ac.uk](mailto:p.sasieni@qmul.ac.uk); [r.greasley@shu.ac.uk](mailto:r.greasley@shu.ac.uk); [d.morrissey@qmul.ac.uk](mailto:d.morrissey@qmul.ac.uk); [Mike.Loosemore@eis2win.co.uk](mailto:Mike.Loosemore@eis2win.co.uk); [abigail.fisher@ucl.ac.uk](mailto:abigail.fisher@ucl.ac.uk); [heather_payne@blueyonder.co.uk](mailto:heather_payne@blueyonder.co.uk); [s.j.c.taylor@qmul.ac.uk](mailto:s.j.c.taylor@qmul.ac.uk); [d.j.rosario@sheffield.ac.uk](mailto:d.j.rosario@sheffield.ac.uk)

PANTERA: online Appendix 1

**Methods**

Semi-Structured interviews

Twelve men who had participated in the exercise arm of the trial were identified by the physiotherapist running the exercise trial to participate in a semi-structured interview with another member of the research team once they had completed the 12 month trial. The identified men were then contacted by a member of the research team, to organise an appropriate time and date to carry out a telephone or face to face interview to share their experiences of the 12 month intervention retrospectively.

All the interviews took place over the telephone (11) or face to face (1), lasted around 25-45 minutes and all men were reminded at the start of the interview that the interview would be recorded. The member of the research team carrying out the interviews had access to adherence rates to the supervised exercise and the independent exercise, this was discussed during the interviews. These interviews used a topic guide (see page 6) and covered *inter alia* reasons for adherence to the trial, experience of the exercise trial, experience of behaviour change support during, any specific benefits due to a results of participating and recommendations for future clinical trial.

**Analysis**

Semi-Structured interviews

Data were analysed using a thematic framework approach (Ritchie & Lewis, 1994), with the aid of NVIVO software. Transcripts were double coded by study researchers (RT and RG). Key themes and sub-themes were identified.

**Results**

Four superordinate themes and twelve subthemes were identified (see Table 4) following the themtical analysis of the semi-structured interview transcripts. These themes are discussed and complemented by selected quotes.

***Motivations for participation in the trial***

Being diagnosed with prostate cancer and being approached to take part in this trial was an incentive for taking part. It was initially discussed with the men that this trial may be of some benefit to their disease and other aspects of their health, the men felt that participation would help them achieve these suggested benefits.

*“Well anything that would help you in a desperate situation like having cancer, I mean I appreciate that I’m a low risk at the moment. That’s the most, that’s all the motivation I needed really. And then as you get into it you realise that it’s therapeutic in all sorts of ways.” 248*

*Well he told me I’d got this stage one prostate cancer, and that by doing exercises it would help reduce it, reduce the PSA, so that’s why I was involved with it. 159*

However, as the trial progressed and as some men didn't see a dramatic change in their PSA, they found this to be 'disheartening' but felt they wanted to continue due to other benefits of the exercise they were experiencing.

*"Whether or not it’s going to help the prostate, the PSA thing, I don’t know, but I’ll just carry on doing it for personal reasons." 167*

Being a part of a research trial and potentially having the opportunity to help others via this trial was something most of the men had considered in their reasons for participation.

*"Yeah, it’s good to be involved in the research side and do something else within that as well, if something came up. Anything can help really, and to help me as well." 205*

*“Well, I do believe that, you know, that people should, should do trials and was quite willing to do trials if it’s going to help myself and mainly if it’s going to help others in the future.” 469*

It was described as an opportunity to improve their lifestyle and get 'fit', as some of the men discussed that they had felt 'unfit' previously.

*" I wanted to get fit, because I was very unfit." 205*

*"Around about the time I was diagnosed with prostate cancer I had a number of, other health issues, I’d had a heart attack a few weeks before being diagnosed with prostate cancer and it was clear from my GP also that I was not managing my diabetes well. But putting the three things together, sort of gave me a massive wake-up call and, and I thought, well, um, if I, if I do, a structured course of exercise over 12 months, which was what the programme was offering me, really I had nothing to lose." 442*

Participation was not without initial concerns, with a 'P.E. lesson' type programme being initially expected. However, due to sufficient pre-trial information, the ability to speak to the research team and being supervised by a physiotherapist, these concerns became less of a worry.

*"It was something, I hadn’t gone to a gym before, so I got a bit worried at the first couple of sessions, but I was all right then." 159*

*"I didn’t know what to expect to be honest. I didn’t know whether it would be, somebody say right do 50 sit ups and 20 press ups. So no, I didn’t know what to expect. But once I saw the gym and attended a couple of times I knew what to expect and I knew that they were checking your pulse rate every five minutes, and asking you what level you felt as far as the amount of work you were doing." 310*

***Adherence***

The trial demonstrated excellent adherence rates (94%) and the reasons for the men's adherence was discussed in great depth. They reported that supervision and having behaviour change support integrated into the supervision was a crucial factor in adherence.

*"I think a combination. It does make you more likely to attend, and also gives you a bit more confidence that somebody’s looking over you if you like." 248*

*"If I’d been left unsupervised I probably would have kept on the same levels of machinery; whereas he urged us to go forward a bit higher each time. And we got on quite well, he was very good."159*

Some of the men had previous experience of other exercise referral schemes such as cardiac rehabilitation, which lacked behaviour change support, with the men stating why this trial was different and more engaging than previous experiences.

*"Well, yeah, I have to say the whole programme was based around goal setting, because not only did we do the exercise in the gym, but we also sat with the specialist from time to time and reviewed our, or set our goals and reviewed our progress towards meeting those goals and we talked about rewards and all that kind of thing, which never happened on the initial cardio programme." 202*

When the men experienced days where they didn't feel like attending, the social aspects and the small amount of time it took out of their day, with allowance for flexibility were the main drivers for attending.

*"Yeah, you know what it’s like. Sometimes you think oh god, do I have to go all the way down there? And then you think well it’s only a couple of hours out of your day. So it’s not a major issue. So once you’re down there it was very sociable and I quite enjoyed it really." 205*

*"The, thought that other people on the group were probably going to be there and that I was missing out." 442*

*"Yeah, yeah and like I said if anything else happened I’d, there were always alternative times, so if ought happened in the morning and I weren’t able to go, there was always another, he’d always got another alternative for you.." 202*

The benefits that the men experienced from participating encouraged adherence, these benefits included the management of comorbidities such as diabetes. Improvement in psychological health and enjoyment were also amongst reasons for adherence.

*"Um, my, er, my diabetes is under control and my GP has said to me if I continue like this I could actually reverse it." 442*

*"Yeah I did, I think it does help with mental issues anyway. People with depression and things like that I think it helps. And I think a lot of people were cheesed off with it, with prostate cancer." 205*

***The trial***

Practicalities of the trial were discussed. The majority of the men though the duration and frequency of the trial was sufficient, with some stating they would have continued having supervised sessions longer than 12 months due to benefit and enjoyment. The delivery sites were seen as being convenient to men; however, parking was sometimes an issue.

*"Actually it all right, it went very quickly. And had it said do another six months or another year I’d have done it. Yeah, because I was enjoying it and it was somewhere, I mean once you’re retired you look forward to going to certain places, and that’s what I did. I enjoyed it."159*

The intensity of the trial was found to gradually increase, with the men becoming fitter, ability to endure more with encouragement and support. Previous experiences of other exercise programmes hadn't allowed for the men to build up their intensity and progress.

*"No, it was about right. I mean I did start doing a bit more as I went on, and obviously you improve over that period of time anyway so you go a bit faster or work a bit harder. So yeah, I found it quite within my capabilities."310*

*"The specialist very carefully monitored my progress and when he felt that I was sort of cruising on a particular exercise, he either upped the effort level or the speed with which I carried that exercise out. That didn’t seem to happen so much on the, on the initial cardio programme. It was just about, getting, um, into the habit of doing some structured exercise. Um, it was like two minutes here and two minutes there and two minutes there, so it, it didn’t, any exercise was not really allowed to be built up to any form of intensity, whereas on the PANTERA programme it was a different kettle of fish." 442*

The use of a watch to monitor independent activity levels produced a mixed response. One view was that men felt frustrated with the use of the watch, found it difficult and invasive to use, also the men reported forgetting to wear it during exercise. Additionally, some of the men felt disappointed that their daily activities such as gardening would not be considered intense enough for their independent exercise.

*"Yeah, my generation are being left behind with technology basically. And even if it’s just a simple question of pressing the right button, you don’t always find it straightaway. But essentially I mean I would say I was 90-95% of the time it’s fine. " 248*

*"Oh, I continue to be frustrated by the necessity to record specific exercise periods outside the supervised sessions . . . Now Um, and, and a period of vigorous gardening, mowing the lawn, raking or digging may in fact be of great benefit, but it, this too is lost. "507*

However, the other view of men found the watch useful as they were able to realise how to successfully exercise to a moderate intensity.

*"I didn’t have anything to tell me what my heart rate was, so I thought I was walking well. And once I’d got that wristband on which told me my heart rate, I realised I wasn’t walking fast enough. I was just strolling probably. So with that wristband on I realised I’d got to work harder. And that’s done me a lot of good that." 159*

***Impact***

*"I have to say the, the programme; the PANTERA programme’s transformed my life" 442*

The men discussed how the programme had impacted upon their quality of life, fitness levels, physical and mental health. Men reported better improved fitness, ability to engage in everyday physical tasks without struggling as much as before and an improved mental health.

*There’s a few, I mean obviously physically I think I’m fitter than I’ve ever "been! Um, so I think it’s been, you know, health, health wise in that just feeling better and feeling fitter has been good and I think there is a bit of a, it’s quite supportive to go to something with other people who are, who have got the same issues." 447*

*"It, it’s really about you being in control of the disease and not letting it control you really in terms of your quality of life" 442*

*"a lot of it is down to the fact that I’m exercising regularly. Er, my Hb1 score is hovering around about 50 now and when I started the PANTERA programme it was up in the late 60s, so if I can get it below 50 that’s going to be ideal for me. My cholesterol is below 5. So yeah, everything, er, everything seems to have, er, sort of benefited from the fact I’m, I’m doing this three hours exercise a week and in the grand scheme of things, three hours is not a lot." 442*

*" I know I’d, you know, I’d, I’d wrote some things down as we started the course about losing weight, getting fitter and that and so I were able to, and that’s what happened, you know, lost my weight." 202*

The trial has increased the confidence around exercise in a great way, with the men stating that their confidence levels were low at the start of the trial, leading to initial concerns of injury or lack of ability.

*"I mean I was unfit but it gave me the confidence to do it. And I was thinking, because I was convinced I was going to keel over and have a heart attack or something if I pushed myself." 205*

*"To be honest what the gym did, it gave me, I mean I was unfit but it gave me the confidence to do it." 205*

Reassurance and being supervised by a health professional aided this improvement in confidence of exercising with a condition safely.

*"There’s a worry that such a level of activity will impact ad-adversely on existing afflictions and morbidities. People need to be reassured, you know, that it’s good for you" 167*

Improved exercise levels after the trial was an outcome that most of the men reported; men have joined gyms, engaged in exercise with their partners or family members and bought monitoring apps or watches as well as exercise equipment since the trial. The men report being able to have the confidence to exercise independently but also how to ensure the exercise they are doing is at a moderate intensity.

*"I have joined a gym; I go three times a week now." 159*

*"So yeah, I wouldn’t have done that if I wasn’t on this programme." 167*

*" I do intend to carry on at, at, at the gym, and also, well, probably once a week, because I’m also going to start taking up, um, walking football as, as well, which is the time exercise of being able to do gym work and, er, the Fit Club work and walking football as well as golf, so there’s quite a bit on there" 469*

**References**

Ritchie J, Spencer L. Qualitative data analysis for applied policy research. London: Routledge 1994.

**Semi-structured interviews schedule**

| **Process evaluation of the exercise intervention** |
| --- |
|  |
| - Reasons for participation in the trial - Provisions of pre-trial information - Structure of the supervised exercise sessions - Engaging with the independent exercise sessions - Frequency, intensity and duration of exercise sessions - Communication with exercise specialist - Comparison to a commercial gym environment - Barriers to exercising - Views on the intervention delivery sites - Overall duration of the intervention - Support from peers/family - Comparison to any experience with conventional support groups - Burden of trial assessments - Relationship with the exercise professional delivering the trial - Perception’s of education around components of exercise behaviour and monitoring - Perceptions of the behaviour change support - Recommendations for the design of future exercise interventions |
